# Corticosterone rapidly increases thorns of CA3 neurons via synaptic/extranuclear glucocorticoid receptor in rat hippocampus

**DOI:** 10.3389/fncir.2013.00191

**Published:** 2013-11-27

**Authors:** Miyuki Yoshiya, Yoshimasa Komatsuzaki, Yasushi Hojo, Muneki Ikeda, Hideo Mukai, Yusuke Hatanaka, Gen Murakami, Mitsuhiro Kawata, Tetsuya Kimoto, Suguru Kawato

**Affiliations:** ^1^Department of Biophysics and Life Sciences, Graduate School of Arts and Sciences, The University of TokyoTokyo, Japan; ^2^Bioinformatics Project of Japan Science and Technology Agency, The University of TokyoTokyo, Japan; ^3^Department of Physics, College of Science and Technology, Nihon UniversityChiyoda, Tokyo, Japan; ^4^Department of Anatomy and Neurobiology, Kyoto Prefectural University of MedicineKamigyo, Kyoto, Japan

**Keywords:** corticosterone, hippocampus, kinase, thorn, stress, spine

## Abstract

Modulation of synapses under acute stress is attracting much attention. Exposure to acute stress induces corticosterone (CORT) secretion from the adrenal cortex, resulting in rapid increase of CORT levels in plasma and the hippocampus. We tried to test whether rapid CORT effects involve activation of essential kinases as non-genomic processes. We demonstrated rapid effects (~1 h) of CORT on the density of thorns, by imaging Lucifer Yellow-injected neurons in adult male rat hippocampal slices. Thorns of thorny excrescences of CA3 hippocampal neurons are post-synaptic regions whose presynaptic partners are mossy fiber terminals. The application of CORT at 100, 500, and 1000 nM induced a rapid increase in the density of thorns in the stratum lucidum of CA3 pyramidal neurons. Co-administration of RU486, an antagonist of glucocorticoid receptor (GR), abolished the effect of CORT. Blocking a single kinase, including MAPK, PKA, or PKC, suppressed CORT-induced enhancement of thorn-genesis. On the other hand, GSK-3β was not involved in the signaling of thorn-genesis. Blocking AMPA receptors suppressed the CORT effect. Expression of CA3 synaptic/extranuclear GR was demonstrated by immunogold electron microscopic analysis. From these results, stress levels of CORT (100–1000 nM) might drive the rapid thorn-genesis via synaptic/extranuclear GR and multiple kinase pathways, although a role of nuclear GRs cannot be completely excluded.

## INTRODUCTION

Functions and architectures of mammalian hippocampus are altered or modulated under the stressful conditions. At least in part, the influences of stress are elicited by corticosterone (CORT), produced in adrenal cortex in response to stress. The hippocampus, center for learning and memory, is particularly sensitive to CORT (Woolley et al., 1990b; Watanabe et al., 1992; Reagan and McEwen, 1997), because glucocorticoid receptors (GR) are abundantly expressed in the hippocampus (Morimoto et al., 1996). The chronic stress-induced increase in CORT slowly produces neuronal cell damage in the hippocampus. Rats exposed to restraint stress for 3 weeks have exhibited neuronal atrophy and decreases of dendritic branches similar to that seen in rats treated with a high dose of CORT for 3 weeks (Woolley et al., 1990b; Watanabe et al., 1992).

The CA3 is considered as a region where controls associative memory (Morris et al., 1982; McNaughton and Morris, 1987). In the stratum lucidum of the CA3, pyramidal neurons have huge and complex post-synaptic structures, named thorny excrescences. One thorny excrescence consists of multiple heads named thorns with one neck along a dendritic branch (Amaral and Dent, 1981; Chicurel and Harris, 1992). One mossy fiber terminal of dentate granule cells contacts multiple thorns of thorny excrescences of CA3 neuron. Thorny excrescences may play essential roles in hippocampal function. Chronic restraint stress has induced retraction of thorny excrescences, which has subsequently been reversed after water maze training. On the other hand, water maze training alone has increased the volume of thorny excrescence as well as the number of thorns per thorny excrescence (Stewart et al., 2005). These slow steroid effects may be mediated by nuclear receptors. Upon binding of steroids to nuclear GR, GR forms dimer and bind to the glucocorticoid response element of genes, resulting in modulation of protein synthesis.

The neuronal response to acute stress (within a few hours) may be very different from that of chronic stress (Sorrells et al., 2009). CORT modulates rapidly (within 2 h) the neuronal activity, which may occur independently of the regulation of the gene expression (Lupien and McEwen, 1997). Stress levels (500–1000 nM) of CORT have been demonstrated to rapidly suppress within 0.5 h the long-term potentiation (LTP) induced by primed burst stimulation (Diamond et al., 1992) or tetanic stimulation (Shibuya et al., 2003). A 0.5 h application of 1–10 μM CORT has rapidly suppressed the *N*-methyl D-aspartate (NMDA)-induced Ca^2^^+^ elevation in the CA1 region of adult hippocampal slices (Sato et al., 2004). In our early study (Komatsuzaki et al., 2012), we demonstrated in CA1 that the application of CORT at 100–1000 nM induces a rapid (~1 h) increase in the density of spines of pyramidal neurons. Blocking kinases, including MAPK, PKA, and PKC, suppressed CORT-induced enhancement of spinogenesis. The receptor of this rapid CORT reaction is synaptic GR.

Compared to the CA1 region, little is known about the response of CA3 hippocampal thorns to the acute stress. We perform the investigations in order to examine the hypothesis that CORT may induce activation of synaptic/extranuclear GR, leading to activation of essential kinases, resulting in rapid remodeling of thorns in CA3 neurons.

## MATERIALS AND METHODS

### ANIMALS

Male Wistar rats were purchased from Saitama Experimental Animal Supply (Japan). All animals were maintained under a 12 h light/12 h dark exposure and free access to food and water. The experimental procedure of this research was approved by the Committee for Animal Research of the University of Tokyo.

### CHEMICALS

Corticosterone, actinomycin D, cyano-nitroquinoxaline-dione (CNQX), MK-801, PD98059, RU486, and Lucifer Yellow CH were purchased from Sigma (USA). Chelerythrine and glycogen synthase kinase-3β (GSK-3β) inhibitor VIII (AR-A014418) were purchased from Calbiochem (Germany). H-89 was purchased from Biomol (USA).

### SLICE PREPARATION

Twelve weeks male rats were deeply anesthetized and decapitated between 9:00 AM and 10:00 AM when plasma CORT levels are low. Immediately after decapitation, the brain was removed from the skull and placed in ice-cold oxygenated (95% O_2_, 5% CO_2_) artificial cerebrospinal fluid (ACSF) containing (in mM): 124 NaCl, 5 KCl, 1.25 NaH_2_PO_4_, 2 MgSO_4_, 2 CaCl_2_, 22 NaHCO_3_, and 10 D-glucose (all from Wako); pH was set at 7.4. Hippocampal slices, 400 μm thick, were prepared with a vibratome (Dosaka, Japan). These slices were “freshly prepared” slices without ACSF incubation. Slices were then incubated for recovery in oxygenated ACSF for 2 h (slice recovery process) in order to obtain conventional “acute slices.” These “acute” slices were then incubated at room temperature with CORT or other drugs such as kinase inhibitors. Immediately after drug exposure (for 0.5, 1, or 2 h), slices were prefixed with 4% paraformaldehyde at 4°C for 2–4 h.

### CURRENT INJECTION OF LUCIFER YELLOW

Thorn imaging and analysis with confocal microscopy was performed essentially as described previously (Komatsuzaki et al., 2005, 2012; Tsurugizawa et al., 2005; Mukai et al., 2007). Briefly, neurons within slices were visualized by an injection of Lucifer Yellow under a Nikon E600FN microscope (Japan) equipped with a C2400–79H infrared camera (Hamamatsu Photonics, Japan) and with a 40× water immersion lens (Nikon). Dye was injected with a glass electrode whose tip (tip diameter < 1 μm) was filled with 5% Lucifer Yellow under a negative DC current of 10 nA for 15 min, using Axopatch 200B (Axon Instruments, USA). Approximately five neurons within a 100–200 μm depth from the surface of a slice were injected (Duan et al., 2002). After labeling, slices were fixed again with 4% paraformaldehyde at 4°C overnight.

### CONFOCAL LASER SCAN MICROSCOPY AND ANALYSIS

The imaging was performed from sequential z-series scans with confocal laser scan microscope (LSM5; Carl Zeiss, Germany) at high zoom (×3.0) with a 63× water immersion lens, NA 1.2. For Lucifer Yellow, the excitation and emission wavelengths were 488 and 515 nm, respectively. Three-dimensional image was reconstructed from approximately 40 sequential z-series sections of every 0.45 μm. The applied zoom factor (×3.0) yielded 23 pixels per 1 μm. The confocal lateral resolution was approximately 0.26 μm. Our resolution limits were regarded to be sufficient to allow the determination of the density of thorns. Confocal images were then deconvoluted using AutoDeblur software (AutoQuant, USA).

In each slice, two to three neurons with more than 100 thorns were analyzed, and at least 90 thorns were counted on each frame. In total, *N* = 12 neurons and *n* = 1400–1800 thorns were analyzed for each drug treatment. The density of thorns was analyzed with Spiso-3D developed by Bioinformatics Project of Kawato’s group (Mukai et al., 2011; Komatsuzaki et al., 2012). Results obtained by Spiso-3D are similar to those by Neurolucida (MicroBrightField, USA) within assessment difference of 2%, and Spiso-3D considerably reduces human errors and experimental labor of manual software (Mukai et al., 2011). The apical dendrite in the stratum lucidum has thorns. Such a dendrite (primary or secondary dendrite) is present within 100 μm from the soma. The density of thorns was calculated from the number of thorns along the dendrite having a total length of 30–100 μm. While counting the thorns in reconstructed images, the position and verification of thorns were aided by three-dimensional reconstructions and by observation of the images in consecutive single planes.

### POSTEMBEDDING IMMUNOGOLD METHOD FOR ELECTRON MICROSCOPY

Immunoelectroscopic analysis was performed essentially as described elsewhere (Hojo et al., 2004; Mukai et al., 2007; Ooishi et al., 2012b). Rat hippocampus was frozen and sliced coronally. Freeze substitution and low-temperature embedding of the specimens was performed as described previously (Roberson et al., 1999). The samples were immersed in uranyl acetate in anhydrous methanol (-90°C). The samples were infiltrated with Lowicryl HM20 resin (Electron Microscopy Sciences, USA) and polymerization was performed with ultraviolet light. Ultrathin sections were cut using a Reichert-Jung ultramicrotome. For immunolabeling, sections were incubated with primary antibody for GR (Morimoto et al., 1996; diluted to 1/3000) overnight, and incubated with secondary gold-tagged (10 nm) Fab fragment in Tris buffered saline (TBS). Sections were counterstained with 1% uranyl acetate, and viewed on a JEOL 1200EX electron microscope (Japan). Images were captured using a CCD camera (Advanced Microscopy Techniques, USA). The antibody is specific to GR in the hippocampus as shown with Western blot (Komatsuzaki et al., 2005; Ooishi et al., 2012b).

### STATISTICAL ANALYSIS

All the data are expressed as means ± SEM. The significance of CORT or drug effect was examined using the Tukey–Kramer *post hoc* multiple comparisons test when one way ANOVA tests yielded *p* < 0.05.

## RESULTS

**FIGURE 1:**
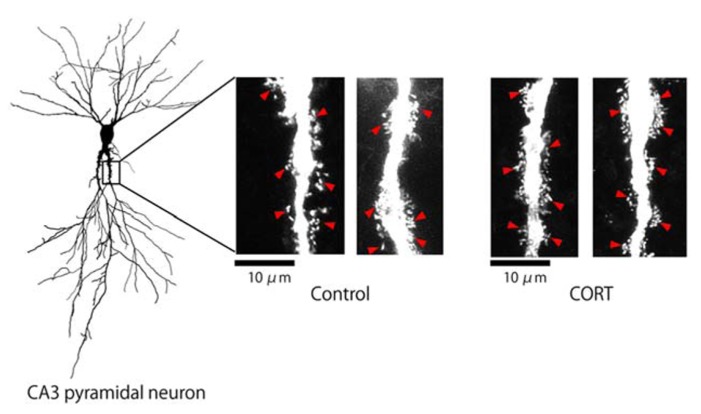
**Changes in the density of thorns by CORT in hippocampal slices.** Maximal intensity projections onto XY plane from z-series confocal micrographs, showing thorns along the primary dendrites of hippocampal CA3 pyramidal neurons. Left image shows a traced whole image of Lucifer Yellow-injected CA3 neuron. Right images show thorns (red arrowheads) without drug-treatments (Control) or thorns after 1 μM CORT treatments (CORT) for 1 h. Bar 10 μm.

We investigated the effect of CORT on the modulation of the thorn density in the hippocampus CA3 stratum lucidum. Lucifer Yellow-injected neurons in hippocampal slices from 12-week-old male rats were imaged using confocal laser scan microscopy (**Figure 1**). Thorny excrescences were located on apical dendrites within 100 μm from the soma, on which mossy fiber terminals attached.

### CORT INCREASED THE DENSITY OF THORNS IN CA3 STRATUM LUCIDUM

**FIGURE 2:**
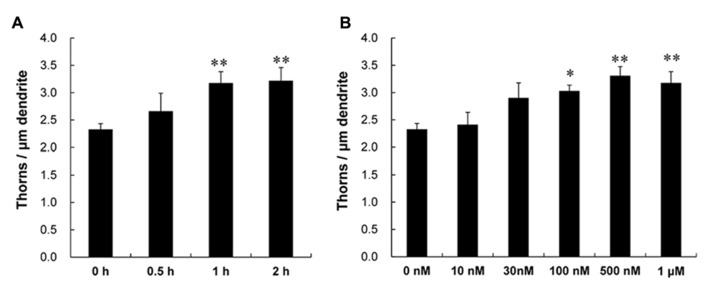
**Time dependency and dose dependency of CORT effects on the thorn density of CA3 neurons.** Thorns were analyzed along the primary and secondary dendrites of pyramidal neurons in the stratum lucidum of CA3 neurons. **(A)** The time dependency of CORT effects on the thorn density in CA3 neurons, after 0.5 h treatment (0.5 h), 1 h treatment (1 h), and 2 h treatment (2 h) in ACSF with 1 μM CORT. As a control, no treatment with CORT (0 h) is shown. **(B)** Dose dependency of CORT treatments on the thorn density. A 1 h treatment in ACSF without CORT (0 nM), with 10 nM CORT (10 nM), with 30 nM CORT (30 nM), with 100 nM CORT (100 nM), with 500 nM CORT (500 nM), and with 1 μM CORT (1 μM). Vertical axis is the average number of thorns per 1 μm of dendrite. Results are reported as mean ± SEM. The significance of CORT or drug effect was examined using the Tukey–Kramer *post hoc* multiple comparisons test when one way ANOVA tests yielded *P* < 0.05. The significance yielded **P < 0.01 *P < 0.05, **P < 0.01 to 0 h and 0 nM. For each drug treatment, we investigated 3 rats, 6 slices, 12 neurons, 12 dendrites, and 1400–1800 thorns.

Following a 1 h treatment with CORT, treated dendrites had significantly more thorns than control dendrites (i.e., 1 h incubation in ACSF without CORT). Time dependency was examined by treating slices for 0.5, 1, and 2 h with 1 μM CORT. The enhancing effect on the total thorn density was approximately proportional to the incubation time, showing 2.7 (0.5 h), 3.2 (1 h), and 3.2 thorns/μm (2 h) in CORT-treatments (**Figure 2A**). Dose dependency was also examined after a 1 h incubation (**Figure 2B**). In CORT-treatment group, the enhancing effect was significant at 1 μM CORT (3.2 thorns/μm) compared with 10 nM (2.4 thorns/μm), 30 nM (2.9 thorns/μm), 100 nM (3.0 thorns/μm), and 500 nM (3.3 thorns/μm) CORT. Because a 1 h treatment with 1 μM CORT was most effective for thorn-genesis, these incubation time and concentration were used in the following investigations unless specified.

**FIGURE 3:**
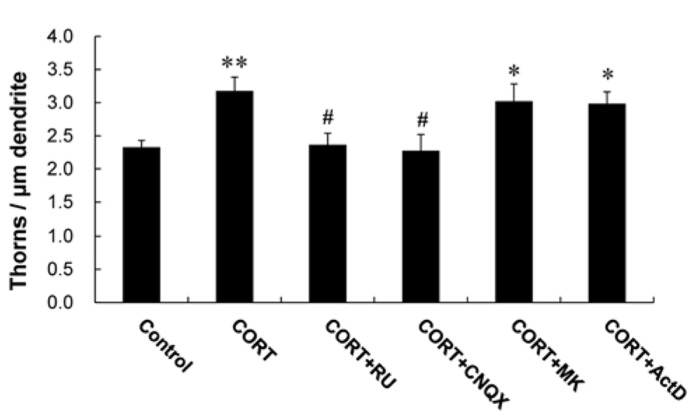
**Effects of blockers of receptors on CORT-induced changes in the thorn density.** A 1 h treatment in ACSF without drugs (Control), with 1 μM CORT (CORT), with 1 and 10 μM RU486 (CORT + RU), with 1 μM CORT and 20 μM CNQX (CORT + CNQX), and with 1 μM CORT and 50 μM MK-801 (CORT + MK). Vertical axis is the average number of thorns per 1 μm of dendrite. Results are reported as mean ± SEM. The significance of CORT or drug effect was examined using the Tukey–Kramer *post hoc* multiple comparisons test when one way ANOVA tests yielded *P* < 0.05. *P < 0.05, **P < 0.01 vs. Control. ^#^*P* < 0.05 vs. CORT. For each drug treatment, we investigated 3 rats, 6 slices, 12 neurons, 12 dendrites, and 1400–1800 thorns.

A 1 h treatment with 1 μM CORT was used in the kinase inhibitor investigations unless specified, because 1 μM CORT showed the strongest effects. Blocking of GR by 10 μM RU486 completely abolished the enhancing effect by 1 μM CORT on the thorn density (2.4 thorns/μm; **Figure 3**). It should be noted that rapid CORT effects (within 1 h) did not induce neurodegeneration, as judged from no significant shrinking in dendrite length and atrophy of cell body (data not shown).

### EFFECT OF CORT WAS BLOCKED BY SEVERAL KINASE INHIBITORS

**FIGURE 4:**
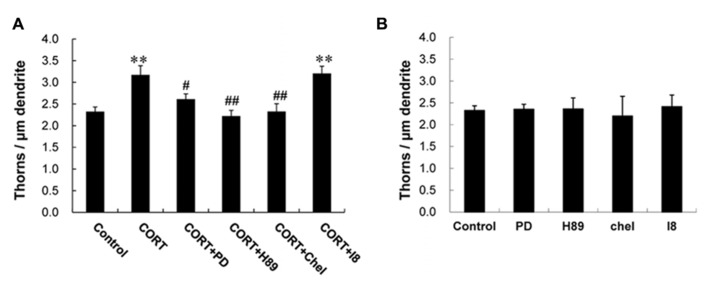
**Suppression effects by kinase inhibitors on CORT-induced changes in the density of thorns.(A)** A 1 h treatment in ACSF without drugs (Control), with 1 μM CORT (CORT), with 1 μM CORT and 20 μM PD98059 (MAPK inhibitor; CORT + PD), with 1 μM CORT and 10 μM H-89 (PKA inhibitor; CORT + H89), with 1 μM CORT and 10 μM chelerythrine (PKC inhibitor; CORT + Chel), and with 1 μM CORT and 10 μM GSK-3β Inhibitor VIII (CORT + I8). **(B)** No effect of kinase inhibitors alone on the density of thorns in CA3 neurons. Abbreviations are the same as **(A)**. Vertical axis is the average number of thorns per 1 μm of dendrite. Results are reported as mean ± SEM. The significance of CORT or drug effect was examined using the Tukey–Kramer post hoc multiple comparisons test when one way ANOVA tests yielded P < 0.05. ^*^^*^P < 0.01 vs. Control. ^#^P < 0.05, ^#^^#^P < 0.01 vs. CORT. For each drug treatment, we investigated 3 rats, 6 slices, 12 neurons, 12 dendrites, and 1600–1800 thorns.

Next we investigated kinase signaling pathways involved in the CORT-induced thorn-genesis using specific inhibitors for kinases (**Figure 4**), by examining the total thorn density. We focus on MAPK, PKA, and PKC, since these kinases often play an important role in synaptic plasticity. Blocking of Erk MAPK by application of 20 μM PD98059 (PD; Dudley et al., 1995), abolished the CORT-induced increase in thorn density resulting in 2.6 thorns/μm. Application of 10 μM H-89 (H89), a protein A kinase inhibitor (Chijiwa et al., 1990), prevented the effect by CORT. Application of 10 μM chelerythrine (Chel), an inhibitor of all the PKC species (alpha, delta, and epsilon; Herbert et al., 1990), prevented the effect by CORT. Blocking of glycogen synthase kinase-3β (GSK-3β) by 10 μM Inhibitor VIII (I8; Bhat et al., 2003) did not alter CORT-induced thorn-genesis. Effect of GSK-3β, which is tau protein kinase, was investigated, since phosphorylation of tau protein (that stabilizes microtubules) is necessary for BDNF (brain-derived neurotrophic factor)-induced spinogenesis in the hippocampus (Chen et al., 2012).

Because the concentrations of inhibitors applied are recommended levels (Bhat et al., 2003; Alonso et al., 2004; Birnbaum et al., 2004; Hammond et al., 2008), the observed inhibitory effects are not artifacts due to excess amount of inhibitors. It should be noted that these kinase inhibitors alone did not significantly affect the thorn density within experimental error, indicating that the observed inhibitory effects are not due to simple blocker’s non-specific suppressive effects (**Figure 4B**).

#### Blocking of glutamate receptors abolished CORT-induced thorn-genesis

We investigated the importance of Ca^2^^+^ homeostasis within thorns on CORT effects. Because the Ca^2^^+^ level may be maintained with spontaneous fluctuation of opening/closing via ionotropic glutamate receptors in thorns, we examined thorn-genesis in the presence of inhibitors of these receptors. 6-cyano-7-nitroquinoxaline-2,3-dione (CNQX), an inhibitor of *α*-amino-3-hydroxy-5-methyl-4-isoxazolepropionate (AMPA) receptor, significantly suppressed the effect of CORT on the thorn density to 2.3 thorns/μm (**Figure 3**). MK-801, an NMDA receptor blocker, did not abolish the CORT effect.

In additional experiments, Actinomycin D (ActD), an mRNA synthesis inhibitor, did not significantly suppress the CORT-induced increase in the density of thorns (**Figure 3**).

#### Ultrastructural analysis for synaptic, extranuclear and nuclear localization of GR

**FIGURE 5:**
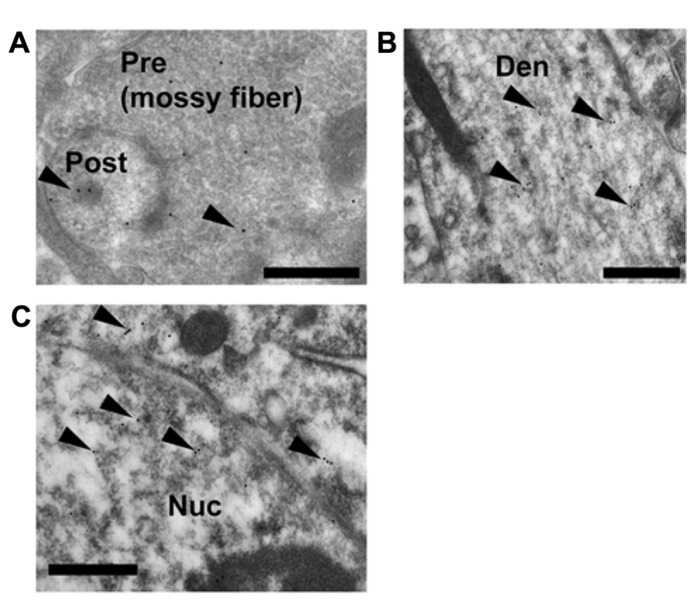
**Immunoelectron microscopic analysis of the distribution of GR within the mossy fiber synapses, dendrites in stratum lucidum and nuclei of pyramidal cells in CA3 region.** Gold particles (arrowheads), specifically indicating the presence of GR, were localized in the pre- and postsynaptic regions **(A)**. In dendrites, gold particles were often found in the cytoplasmic space **(B)**. Gold particles were also localized in the nuclei **(C)**. A search for immuno-gold labeled GR proteins was performed at least 30 synapses at CA3 region from more than 100 independent images. A 1:3000 dilution of IgG was used to prevent non-specific labeling. Pre, presynaptic region; Post, post synaptic region; Den, dendrite; Nuc, nucleus. Scale bar, 500 nm.

To explain the site of rapid thorn-genesis by the activation of GR, a clarification of the subcellular localization (particularly the synaptic or extranuclear localization) of GR in CA3 pyramidal cells is essential. The synaptic, extranuclear and nuclear localization of GR was clarified via ultrastructural investigations using GR IgG (1/3000). An immunoelectron microscopic analysis using post-embedded immunogold was performed to determine the localization of GR-immunoreactivity in the hippocampal CA3 pyramidal cells. GR was localized not only in the nuclei but also in both the axon terminals and thorns of pyramidal cells (**Figure 5A**). At postsynapses, gold particles were distributed within the cytoplasm of the thorn head. Significant labeling along dendrites was also observed (**Figure 5B**). For a search of immunogold-labeled GR proteins, multiple labeling (three or more) of immunogold in the pre- and post-synaptic compartments was confirmed in at least 100 images. Each image contained several synapses among which at least one synapse expressed GR particles. We also observed some synapses in one image that did not express GR particles. Consequently, we observed approximately 10–20% of synapses that expressed GR particles. Preadsorption of the antibody with GR antigen (30 μg/ml) resulted in the disappearance of immunoreactivity.

The antibody used for the current experiments were shown to have specific binding to GR in the hippocampus with Western blotting (Komatsuzaki et al., 2005; Ooishi et al., 2012b). As a negative control of GR specificity, no GR immunoreactivity was observed in the magnocellular division of the paraventricular nucleus (Morimoto et al., 1996).

## DISCUSSION

The current study demonstrated GR- and kinase-dependent mechanisms of rapid CORT-induced thorn-genesis in CA3 pyramidal neurons of the adult male rat hippocampus. An extremely concentrated distribution of thorny excrescences, as compared with sparse distribution of spines located in other regions, such as CA1, prevented detailed analysis of thorny excrescences by previous studies using Golgi staining methods (Gould et al., 1990; Woolley et al., 1990a). We were able to analyze the number of thorns by the high-resolution image analysis of Lucifer Yellow-injected neurons, using deconvolution, and digital three-dimensional analysis. Mossy fiber terminals originating from granule cells in DG provide excitatory inputs to CA3 neurons via thorny excrescences in the stratum lucidum (Amaral and Dent, 1981; Chicurel and Harris, 1992). Our data imply that CORT may rapidly enhance the excitatory input to CA3 from DG by increasing the density of thorns.

### STEROID LEVELS IN “ACUTE” SLICES USED FOR THORN EXPERIMENTS

Following exposure to stress in rat, a high-dose of CORT (about 1 μM) is secreted by the adrenal cortex and readily penetrates into the brain from the blood circulation. The steroid levels in slices used for thorn analysis must be known. From our earlier study (Komatsuzaki et al., 2012), the CORT concentration in the freshly isolated hippocampus was 400–1000 nM as determined by mass-spectrometric analysis (Hojo et al., 2009, 2011; Ooishi et al., 2012a,b), because rats were under decapitation stress which caused penetration of elevated plasma CORT (1-2 μM) into the hippocampus (Pardridge and Mietus, 1979). Note that 1-2 μM CORT is more than the upper limit capacity (400–600 nM) of CORT binding globulin (Breuner and Orchinik, 2002). However, the control “acute” slices, used for the thorn analysis, have very low CORT level of approximaterly 2 nM by recovery incubation of “fresh” slices (with high CORT concentration of 400–1000 nM) in steroid-free ACSF for 2 h, due to leakage of CORT from slices to ACSF (Hojo et al., 2011; Ooishi et al., 2012a,b). This CORT leakage always occurs in hippocampal slices prepared at different time (morning, afternoon, or evening), therefore control acute slices should have always low CORT. From these reasons, the enhanced thorn-genesis occurred upon increase in CORT level from approximately 2 nM (control) to 100–1000 nM by CORT application.

### CONTRIBUTION OF SYNAPTIC/EXTRANUCLEAR GR TO THE RAPID MODULATION

Since the effect of CORT had been blocked by RU486, we confirmed that CORT-effect was directly mediated by GR. The expression of GR in the CA3 region was demonstrated by immunoelectron microscopy, although the GR expression in CA3 was a little bit weak in immunohistochemistry and *in situ* hybridization, in comparison with CA1 (Morimoto et al., 1996). We observed GR localized within the postsynaptic structures via postembedding immunogold staining (**Figure 5**). The current CORT treatment may rapidly activate the synaptic GR. GR was often located in the cytoplasm of thorns (Komatsuzaki et al., 2005; Ooishi et al., 2012b). GR was observed also in dendrites and nuclei (Komatsuzaki et al., 2005; Ooishi et al., 2012b). Our earlier study shows that GR also expressed in purified PSD fraction by Western blot analysis (Komatsuzaki et al., 2005; Ooishi et al., 2012b). These results suggest that the rapid modulation of thorns by CORT may be mediated by postsynaptic or extranuclear GR. The involvement of GR in the CORT effect was also supported by GR antagonist RU486 which blocked CORT-induced thorn-genesis (**Figure 3**). In addition, GR agonist dexamethasone induces rapid spinogenesis in hippocampal CA1 neurons within 1 h (Komatsuzaki et al., 2005). Membrane GR-induced rapid PKA activation (~1 h) has been demonstrated for inhibitory avoidance behavior via rat basolateral amygdala (Roozendaal et al., 2002), suggesting that synaptic GR may activate kinases.

Since in our “acute” slice CORT level is around 2 nM, all mineral corticoid receptor (MR) may be occupied due to high affinity (Kd ~ 0.5 nM) to aldosterone or CORT. Therefore the effect of CORT on thorns is mainly mediated by GR but not by MR, in the presence of 1 μM CORT. The 1 h responses may be too rapid for nuclear GR actions which often need more than 5 h due to genetic processes. As an another possibility, these rapid actions may include rapid genomic actions via nuclear GR, which are suggested as a reduction of dendritic length (1–4 h; Alfarez et al., 2009) or an impairment of enhancement of voltage-dependent Ca^2^^+^ currents in mutated GR (1–4 h; Karst et al., 2000), in hippocampal neurons. However, since ActD did not suppress the CORT-induced thorn-increase (**Figure 3**), rapid transcriptional process probably does not participate in the thorn-genesis. A significant suppression of thorn changes by application of kinase inhibitors (**Figure 4**) may put more weight on GR-kinase pathway rather than DNA binding of GR.

Since RU486 suppresses not only GR but also progesterone receptor (PR) in **Figure 3**, progesterone (PROG) effect should be considered. The treatment of slices with 10 nM PROG for 1 h did not significantly increase the thorn density within experimental error (data not shown), excluding the involvement of PROG and PR in the observed thorn-genesis.

### CORT-INDUCED THORN-GENESIS VIA KINASE NETWORKS AND THEIR DOWNSTREAM

**FIGURE 6:**
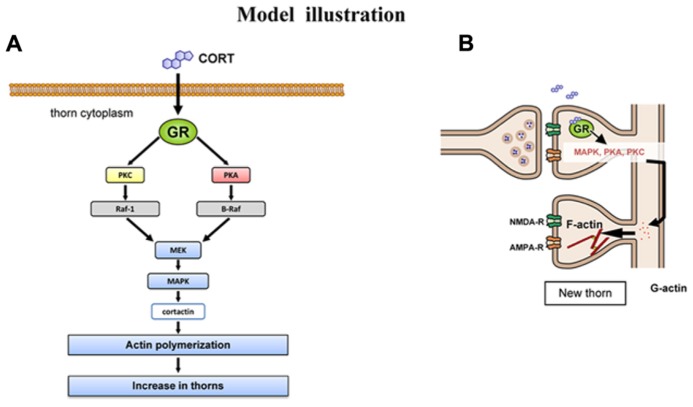
**Model illustration.** **(A)** Schematic illustration of CORT-driven multiple kinase pathways. Upon binding of CORT, GR induces the sequential activation of PKA, PKC, MEK, and Erk MAPK. Erk MAPK regulates phosphorylation of actin-related proteins such as cortactin, resulting in actin reorganization. **(B)** Schematic illustration of CORT-induced rapid thorn-genesis via multiple kinase pathways.

There is increasing evidence implying that CORT is capable of driving rapid signaling (around 1h), independent of slow transcriptional signaling (model illustration in **Figure 6**). Rapid MAPK activation (~2 h) via GR has been demonstrated in the mice hippocampus or pituitary-derived cell-lines AtT20 (Revest et al., 2005). The expression of Raf1, Ras, p-MAPK is elevated rapidly upon application of 10 nM CORT. Fear conditioning of mice is dependent on GR-MAPK pathway. Rapid PKA activation (phosphorylation of PKA; ~1 h) via membrane located GR has been demonstrated in rat basolateral amygdala (Roozendaal et al., 2002).

To consider the molecular mechanisms of kinase signaling in the modulation of CA3 thorn, we temporarily use the model of CA1 region. In this model, MAPK cascade is coupled with PKA and PKC via PKC → Raf1 → MAPK, PKA → B-Raf → MAPK in synaptic modulation (Adams et al., 2000; Komatsuzaki et al., 2012). Taking the knowledge into account, MAPK may be a key kinase responsible for modulation of thorns. The target of Erk MAPK in thorn reorganization is cortactin, since Erk MAPK is known to phosphorylate cortactin, a structural protein associated to actin (MacQueen et al., 2003). Cortactin interacts with both F-actin and actin-related protein (Arp) 2/3 complex as well as scaffold protein Shank in the PSD at the SH3 domain (Weed et al., 1998; Daly, 2004), resulting in promotion of actin fiber remodeling within spines or thorns.

It is thus probable that CORT exerts its effect on thorns via cortactin-actin pathway. Cortactin has multiple phosphorylation sites which are activated by MAPK (Campbell et al., 1999). Phosphorylation of cortactin may promote assembly of actin cytoskeletal matrices, resulting in thorn formation or modulation of thorn morphology (Hering and Sheng, 2003). These sites are putative phosphorylation sites also for other serine/threonine kinase (PKA or PKC) that are activated by CORT.

In the case of *in vivo* hippocampus, the similar rapid CORT-induced modulation of thorns might occur. In response to acute severe stress, elevation of plasma CORT (to 1–2 μM) occurs, resulting in elevation of hippocampal CORT to 0.5–1 μM, due to penetration of CORT into hippocampus after crossing the Blood Brain Barrier (Higo et al., 2011). This increase of CORT level should affect thorn-genesis *in vivo*.

The abolishment of the CORT-induced increase in the density of thorns by CNQX (**Figure 3**) suggests the correlation of the CORT signaling pathway with AMPA receptors. Maintenance of the suitable basal Ca^2+^ level may be important for action of PKC or MAPK on thorn-genesis. CNQX may decrease the basal Ca^2+^ level which is spontaneously formed by ion exchange systems consisting of AMPA receptors plus voltage activated calcium channels. This explanation is deduced from previous study which has shown that the Ca^2+^ influx within thorny excrescences has occurred via AMPA receptor-dependent voltage activated calcium channels during subthreshold activation of CA3 neurons, while NMDA receptors-mediated calcium influx within CA3 thorny excrescences is smaller than that in CA1 spines upon subthreshold activation (Monaghan et al., 1983; Baude et al., 1995; Fritschy et al., 1999; Reid et al., 2001; Reid, 2002).

### OTHER EXAMPLES OF KINASE-DEPENDENT THORN-GENESIS OR SPINOGENESIS

The activation of synaptic androgen receptor AR by testosterone or dihydrotestosterone induces a rapid increase of thorns of thorny excrescences in CA3 pyramidal neurons of adult rat hippocampal “acute” slices within 2 h. The rapid synaptic action of androgen is also mediated by activation of many kinases. This thorn-genesis induced by androgen is mediated by Erk MAPK, p38 MAPK, PKC, CaMKII, but not by PKA and PI3K (Hatanaka et al., 2009). The rapid spinogenesis by estradiol in CA3 and CA1 pyramidal neurons of hippocampal “acute” slices is mediated by synaptic ERα → Erk MAPK pathway (Tsurugizawa et al., 2005; Mukai et al., 2007).

Concerning CA3 rapid stress effects, the administration of corticotropin releasing hormone (CRH), rapidly (within 0.5 h) induces loss of CA3 dendritic spines (different from thorns) in stratum radiatum, using Yellow Fluorescence Protein (YFP)-expressing hippocampal neurons (Chen et al., 2008).

One of the physiological significance of GR-induced increase in thorns may be that upon acute stress (for example, stress at examination or oral interview) neurons may be activated and new thorns may appear, resulting in synaptic remodeling. This is very different from chronic stress-induced depression of neural activities via nuclear GR-induced genetic transcription processes.

## Conflict of Interest Statement

The authors declare that the research was conducted in the absence of any commercial or financial relationships that could be construed as a potential conflict of interest.
